# Single-cell analysis reveals the intra-tumor heterogeneity and identifies MLXIPL as a biomarker in the cellular trajectory of hepatocellular carcinoma

**DOI:** 10.1038/s41420-021-00403-5

**Published:** 2021-01-18

**Authors:** Xiao Dong, Fan Wang, Chuan Liu, Jing Ling, Xuebing Jia, Feifei Shen, Ning Yang, Sibo Zhu, Lin Zhong, Qi Li

**Affiliations:** 1 Department of Oncology, Shanghai General Hospital, Shanghai Jiao Tong University School of Medicine, Shanghai, 200080 China; 2 Department of Hepatic Surgery, Shanghai Eastern Hepatobiliary Surgery Hospital, Second Military Medical University, Shanghai, 200438 China; 3 Center for Pharmacogenomics, School of Life Sciences, Fudan University, Shanghai, 200438 China; 4 Department of Hepatobiliary and General Surgery, Shanghai General Hospital, Shanghai Jiao Tong University School of Medicine, Shanghai, 200080 China

**Keywords:** Prognostic markers, Cancer genomics

## Abstract

Hepatocellular carcinoma (HCC) is a globally prevailing cancer with a low 5-year survival rate. Little is known about its intricate gene expression profile. Single-cell RNA sequencing is an indispensable tool to explore the genetic characteristics of HCC at a more detailed level. In this study, we profiled the gene expression of single cells from human HCC tumor and para-tumor tissues using the Smart-seq 2 sequencing method. Based on differentially expressed genes, we identified heterogeneous subclones in HCC tissues, including five HCC and two hepatocyte subclones. We then carried out hub-gene co-network and functional annotations analysis followed pseudo-time analysis with regulated transcriptional factor co-networks to determine HCC cellular trajectory. We found that MLX interacting protein like (MLXIPL) was commonly upregulated in the single cells and tissues and associated with a poor survival rate in HCC. Mechanistically, MLXIPL activation is crucial for promoting cell proliferation and inhibits cell apoptosis by accelerating cell glycolysis. Taken together, our work identifies the heterogeneity of HCC subclones, and suggests MLXIPL might be a promising therapeutic target for HCC.

## Introduction

Primary liver cancer (PLC) is the seventh most prevalent cancer and the third leading cause of cancer-related death worldwide^1^. Hepatocellular carcinoma (HCC) is the dominant pathological type, which accounts for 75–85% of PLC. HBV, HCV, aflatoxin B1, and alcohol abuse are the major risk factors for HCC^2,3^. Therapeutic strategies have gradually improved the overall survival (OS) rate of HCC patients, but the prognosis is still poor^3,4^. Targeting drugs, such as the multi-tyrosine kinase inhibitors sorafenib and regorafenib, have shown excellent therapeutic outcomes^5,6^. However, HCC patients are prone to suffer intrahepatic tumor recurrence and distant metastasis after surgery^7^. These characteristics of HCC are maybe caused by a small number of tumor cell subpopulations, which carry more aggressive genetic or phenotypic alterations, thereby escaping conventional detection^4^.

Single-cell sequencing is an emerging technology that provides genomic, transcriptomic, and epigenetic information of single cells. It allows heterogeneous cells to be sequenced individually to reveal the unique and subtle changes of the population and facilitates the discovery and definition of new cell subtypes^8^. Researchers have performed single-cell RNA sequencing (scRNA-seq) of liver tissue to construct human liver cell maps^9,10^. In doing so they have found heterogeneity of in the HCC tumor microenvironment (TME) and identified tumor stem cells^11,12^. For example, Ma et al.^11^ found that heterogeneity in malignant liver cells contributes to the diverse landscape of the TME. Ho et al.^13^ identified a CD24+/CD44+ enriched cell subpopulation within EPCAM+ cells, which indicates the presence of a novel stemness-related subclone in HCC.

In this study, we characterize differences between identified subcellular populations and highlight possible functional target genes. First, we identified the unique cell subclones in HCC tumor populations and then measured their corresponding biomarkers using differentially expressed genes (DEG) analysis. Hub-gene network analysis and functional annotations of different subpopulations participating in biological pathways were calculated to reveal their downstream implications. Next, we infer the cellular trajectory, a route depicting normal liver cells and HCC subclones, which may reflect the hepatocellular transition to malignancy. We discover multiple transcription factors (TF) that dominate this transition pathway. Among them, MLX interacting protein like (MLXIPL, Chrebp) was the most remarkable TF, closely associated with the prognosis of HCC. Our subsequent experiments demonstrated that the malignant biological behaviors of MLXIPL were mainly due to an increase in aerobic glycolysis. Thus, our study reveals an integrated transcriptomic landscape and identified a specialized biomarker for the future treatment of HCC.

## Results

### Clinicopathological feature of patients and the filtration of single cells

A total of six HCC patients were recruited and the clinicopathological features are shown in Supplementary Table 1. We obtained single cells of their liver specimens and used fluorescence-activated cell sorting (FACS) analysis to ensure the selection of living cells (Supplementary Fig. 1). After rigorous quality control and step-by-step filtration from 521 libraries, we obtained 405 single cells qualified for further analysis (Supplementary Table 2). A total of 21,459 genes (of 22,336 genes) that passed the filtration were used for analysis in further experiments. The mean number of qualified genes per cell and mean library size were 4939 (from 2000 to 10,891) and 44,408 (from 10,000 to 273,371), respectively (Supplementary Fig. 2A). The number of qualified genes per cell and library size from each patient are shown in Supplementary Fig. 2B, C.

### Cell subpopulations in the livers of HCC patients

**Fig. 1:**
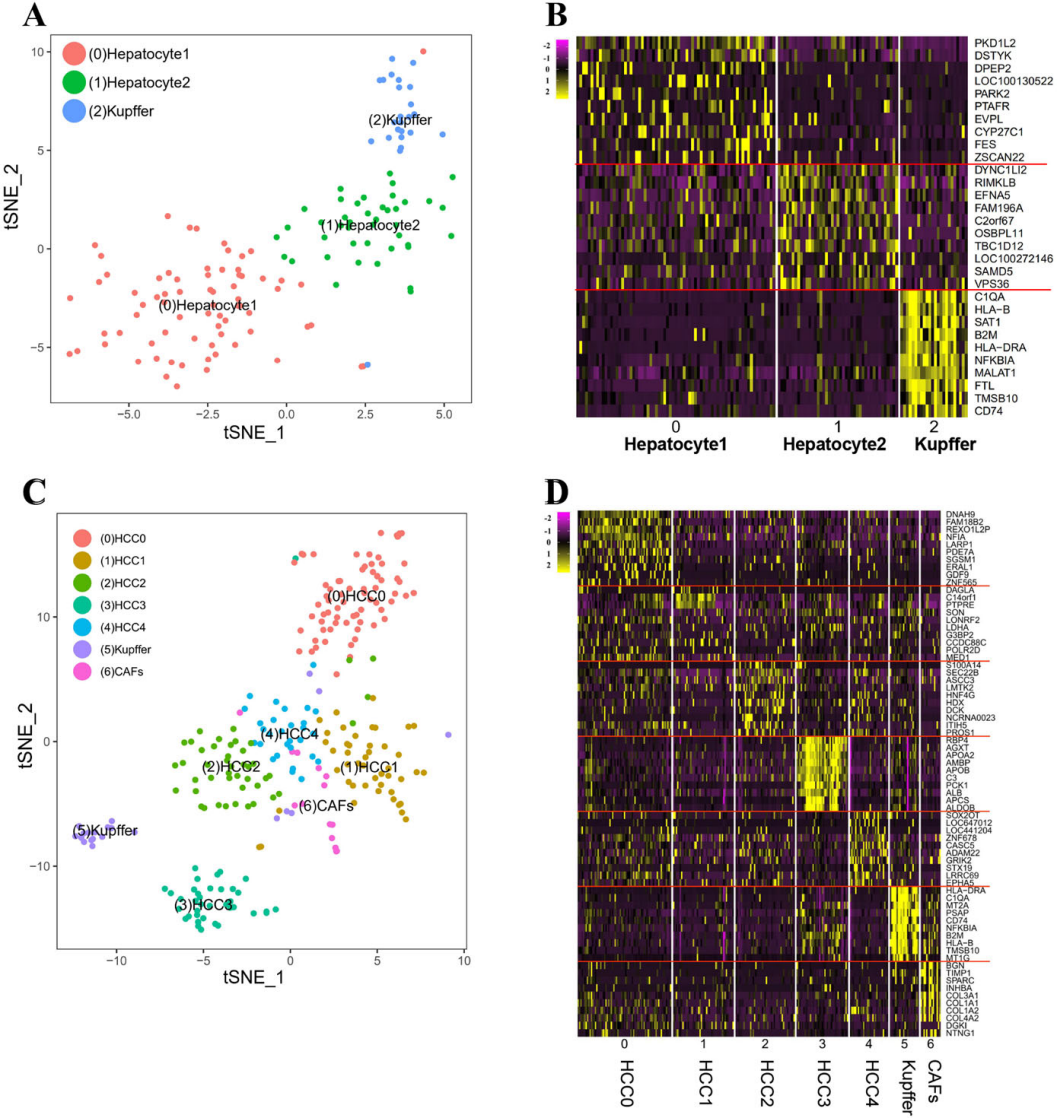
Singles cell subpopulations and specific DEGs identification in HCC tissues. **A** The t-SNE projected three main subclones of single cells clusters from para-tumor tissues. Each cluster was labeled with Arabic number. **B** Heatmap of the normalized top 10 DEGs of single cells clusters in three main subclones from para-tumor tissues. **C** The t-SNE projected seven main subclones of single cells clusters from HCC tissues. **D** Heatmap of the normalized top 10 DEGs of seven main subclones in HCC tissues.

Clustering analysis identified three clusters in para-tumor tissues, including subclones of hepatocyte 1 (cluster 0), hepatocyte 2 (cluster 1), and Kupffer cell (cluster 2) (Fig. 1A). To distinguish the cell phenotypes, we selected the top 10 DEGs from the three identified clusters using a heatmap (Fig. 1B).

The t-distributed stochastic neighbor embedding (t-SNE) plot revealed seven main clusters in the HCC tissues, including five HCC subpopulations (clusters 0–4), a Kupffer cell subpopulation (cluster 5), and a cancer-associated fibroblast subpopulation (CAFs, cluster 6) (Fig. 1C). We then profiled the top 10 DEGs of the identified clusters from HCC tissues using a heatmap (Fig. 1D).

**Fig. 2:**
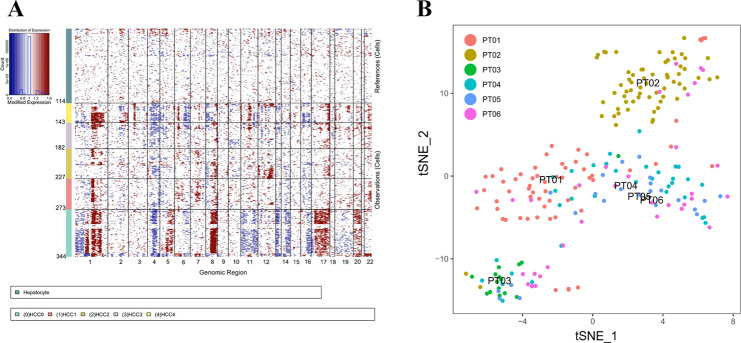
Intra-tumoral heterogeneity at the single-cell transcriptional levels. **A** Chromosomal landscape of copy-number variations (CNV) distinguishes malignant from nonmalignant cells at transcriptomes. Red, amplifications; blue, deletions. **B** A t-SNE plot of all the 405 single cells from six liver cancer patients (patients indicated by colors). Patients’ ID was named as PT0X.

We further discriminated malignant cells from nonmalignant cells by inferring chromosomal copy-number variations (CNV) based on transcriptomes. Chromosomal deletions and amplifications among our cohort are indicated in Fig. 2A, and the results showed the amplifications of chromosomes 1, 8, and 17, and deletions of chromosomes 4, 11, and 16 of HCC single cells (Fig. 2A). The inferred CNV profiles are almost consistent with that in liver cancer from previously published studies^14–16^.

A t-SNE map showed cells clustered according to individual tumor tissues, indicating patient-specific clusters (Fig. 2B). Patient 1 (PT01) was dominated by subpopulation HCC2, and Patient 2 (PT02) was dominated by subpopulation HCC0. When analyzing cell subpopulations in other patients, we cannot identify subpopulations that dominated over others. These results demonstrate the intra-heterogeneity of HCC, as well as the relatively little inter-tumor heterogeneity across patients.

### Gene co-network and functional annotation of HCC single-cell subpopulations

**Fig. 3:**
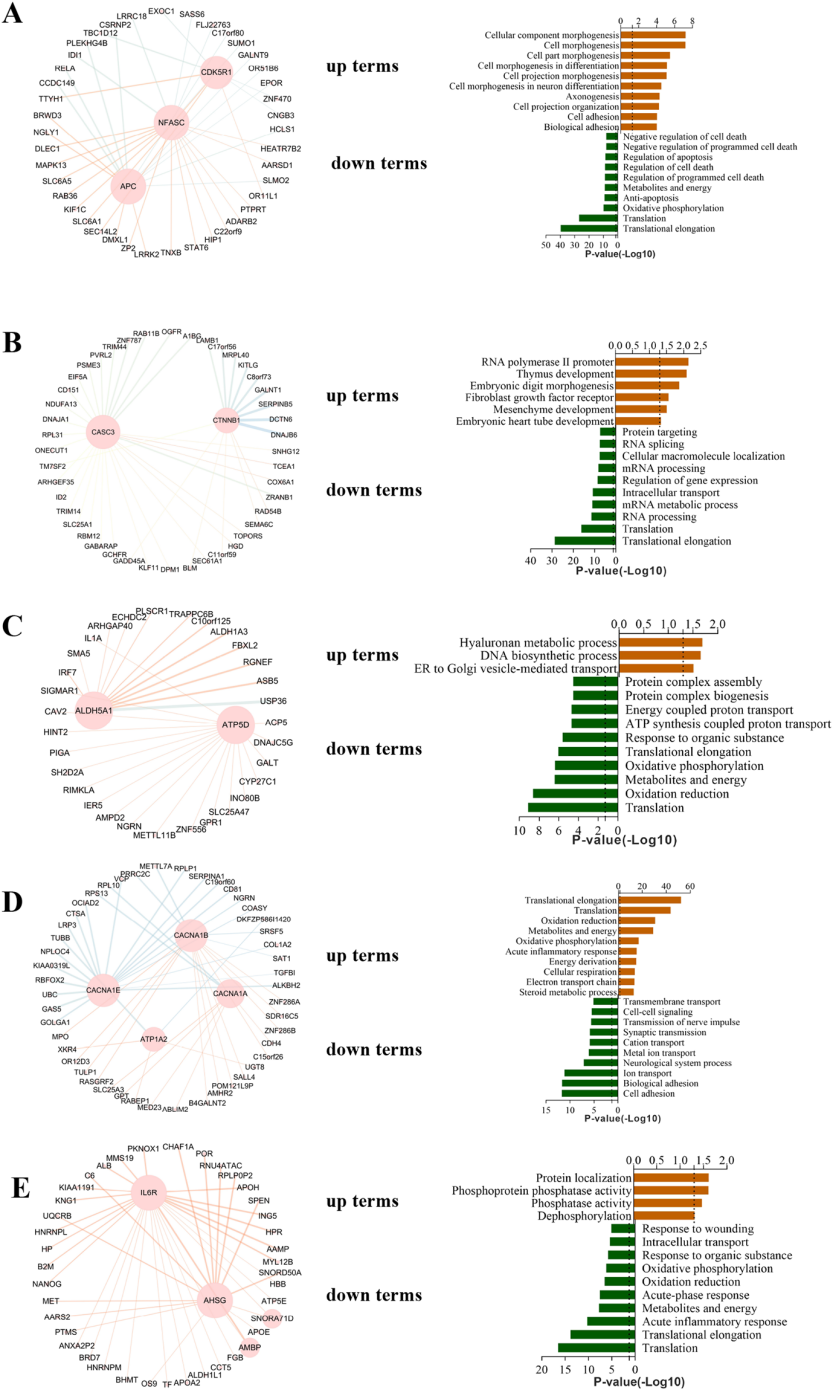
Hub-gene co-network and functional annotation of HCC0–4 (clusters 0–4). **A** Hub-gene co-network and GOBP functional analysis of HCC0 (cluster 0). **B** Hub-gene co-network and GOBP functional analysis of HCC1 (cluster 1). **C** Hub-gene network and GOBP functional analysis of HCC2 (cluster 2). **D** Hub-gene network and GOBP functional analysis of HCC3 (cluster 3). **E** Hub-gene network and GOBP functional analysis of HCC4 (cluster 4).

To detect the functional abnormalities, which would guide the direction of the next biological experiments, we performed co-network and functional annotation analyses based on Gene Ontology Biological Processes (GOBP) and Kyoto Encyclopedia of Genes and Genomes (KEGG) databases. The results showed that genes (CDK5R1, NFASC, APC, and other cluster-specific DEGs) in HCC0 (cluster 0) were enriched. The upregulated genes were related to cell morphogenesis signaling, and the downregulated genes were related to cell death (Fig. 3A and Supplementary Fig. 3A). The results demonstrated that genes (CASC3, CTNNB1, and other cluster-specific DEGs) in HCC1 (cluster 1) were enriched in mesenchyme development, Wnt signaling, and PI3K-Akt signaling. The downregulated genes related to mRNA processing (Fig. 3B and Supplementary Fig. 3B). Genes (ALDH5A1, ATP5D, and other cluster-specific DEGs) in HCC2 (cluster 2) were enriched in threonine phosphorylation and MAP kinase activity, and the downregulated genes related to protein translation and oxidation (Figs. 3C and S3C). Genes (CACNA1A, CACNA1B, CACNA1E, ATP1A2, and other cluster-specific DEGs) in HCC3 (cluster 3) were associated with nutrient metabolism, including lipid metabolism, amino acid metabolism, and energy metabolism disorder (cellular respiration and oxidation phosphorylation) (Fig. 3D and Supplementary Fig. 3D). Thus, we speculated that HCC3 (cluster 3) exhibited widespread metabolic disorders. Genes (IL6R, AHSG, and other cluster-specific DEGs) in HCC4 (cluster 4) were enriched in phosphatase activity and downregulated process related to immune disorders (Fig. 3E and Supplementary Fig. 3E).

### Cellular trajectory characteristics of single HCC cells

**Fig. 4:**
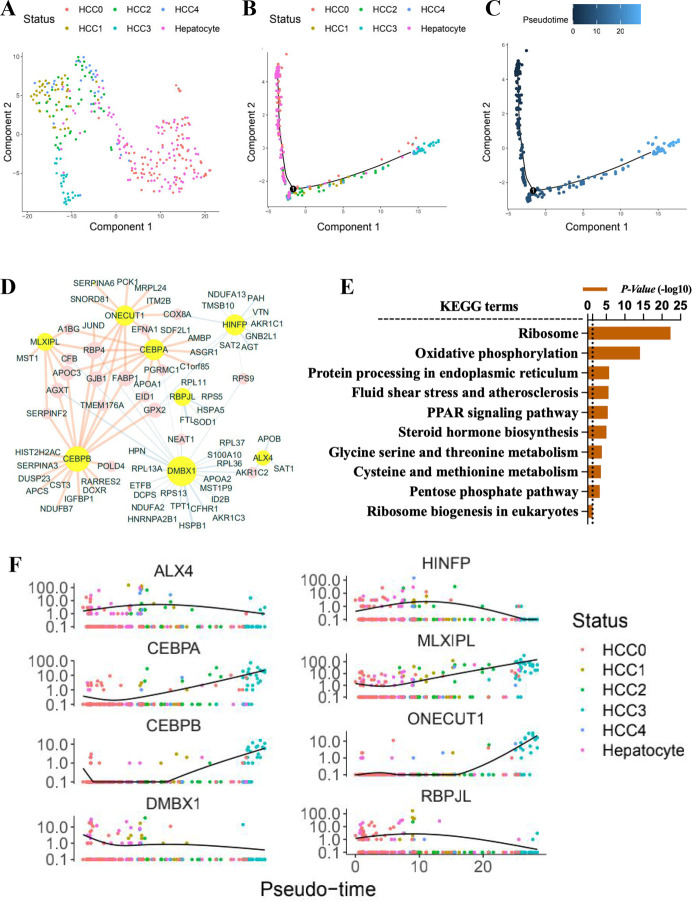
Cellular trajectory pattern and its major driving genes. **A** An unsupervised t-SNE plot showed sporadic distributed five HCC clusters in HCC tissues, and hepatocellular cells in para-tumor tissues. **B** Cellular trajectory of five HCC cells subpopulations and para-tumor hepatocytes. The t-SNE plot revealed the distribution of single cells determined by Monocle. **C** The direction of the cellular trajectory determined by unsupervised pseudo-time. **D** Regulatory co-networks of cellular trajectory-related transcription factors and their regulated genes. Genes in yellow circles were the most critical transcription factors. **E** GOBP functional annotation related to the cellular trajectory. **F** Plots showed the trends of expression profiles of eight key transcription factors in different subclones as pseudo-time.

An unsupervised t-SNE plot revealed the distribution of hepatocyte-derived cells, including HCC0–HCC4 and hepatocytes. To investigate the HCC subpopulations cellular trajectory, we applied Monocle R package approach. We noted that the main cluster of HCC0 and hepatocytes exhibited a highly merged pattern at origination. The HCC single-cell trajectory starts from HCC0 (Fig. 4A). We assumed the path had a tree structure, with a root state of normal hepatocytes and HCC0, and a leave state of other HCC subclones. The end of the main branch is the HCC3 subclone, which is enriched in metabolic disorder-related pathways (Figs. 3D and S3D). Having identified the starting point of differentiation, we elucidated the time of differentiation of each cell (unsupervised pseudo-time). We then obtained the direction of the trajectory in five HCC subclones as an order of HCC0–HCC4 (Fig. 4C).

Based on the pseudo-temporal continuum profile, we identified the TF (ALX4, HINFP, CEBPA, CEBPB, DMBX1, MLXIPL, ONECUT1, and RBPJL) and depicted the regulated kernel genes co-networks (Fig. 4D), which were subjected to the metabolism disorders (Fig. 4E). The plot showed the trends of expression profiles of eight TF in different subclones as pseudo-time (Fig. 4F). The single-cell trajectory-related TF and their regulated genes are listed in Supplementary Table 3. Thus, our work may lead to a comprehensive understanding the cellular metabolism disorders on the paths of subpopulations in HCC.

### MLXIPL is associated with poor prognosis of HCC

**Fig. 5:**
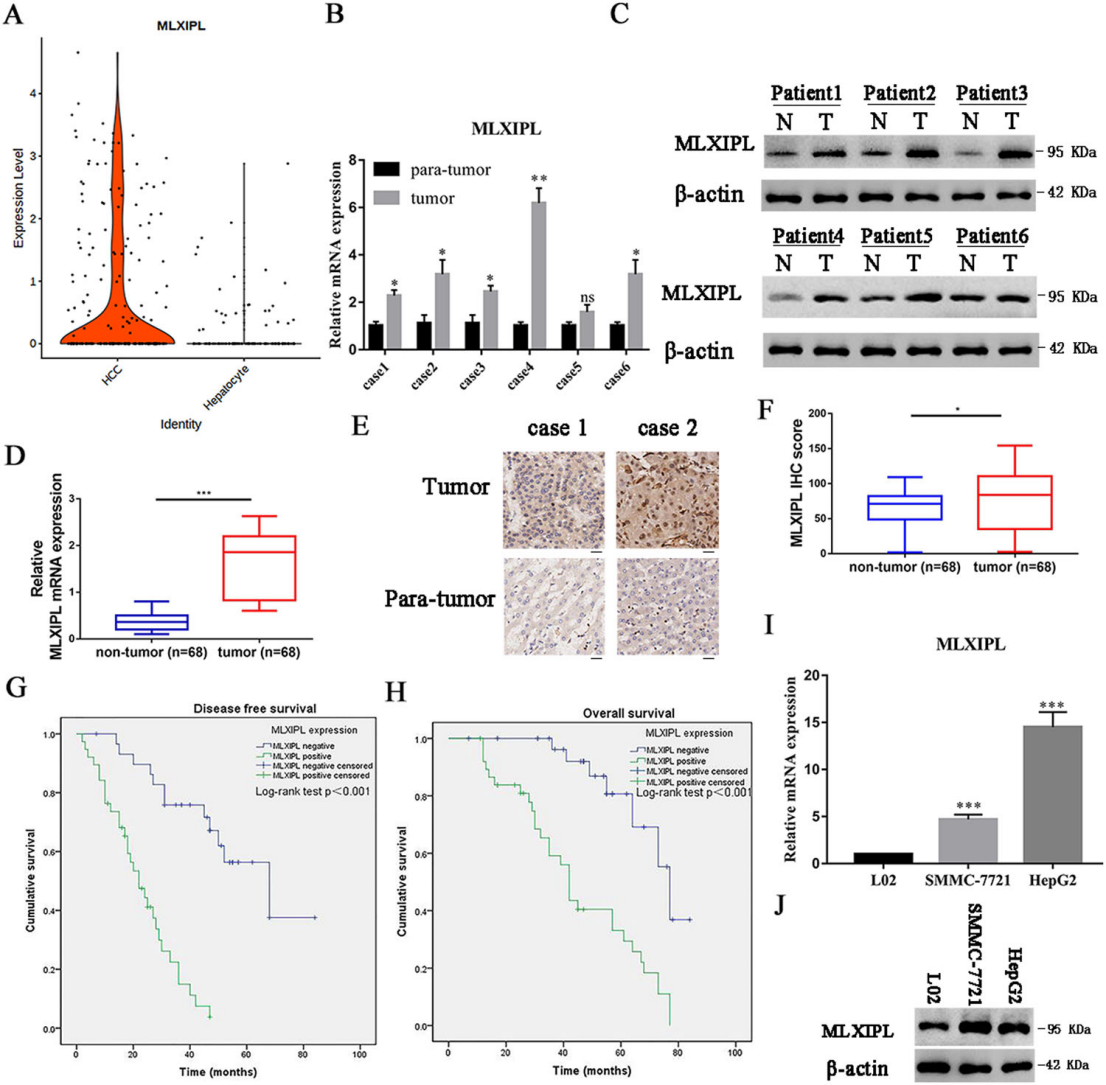
MLXIPL, as a potential biomarker, is correlated with a poor survival rate of HCC patients. **A** A violin plot revealed the mRNA level of MLXIPL in HCC clusters comparing to hepatocyte clusters. **B**, **C** Quantitative real-time PCR and western blotting analysis were performed to detect MLXIPL protein expression in six patients of HCC and para-tumor tissues used in single-cell sequence. ns *P* > 0.05; *P < 0.05; **P < 0.01. **D** PCR analysis were performed to detect MLXIPL mRNA expression in 68 patients of HCC and para-tumor tissues used in single-cell sequence. ***P < 0.001. **E**, **F** Representative immunohistochemistry pictures and IHC score of the MLXIPL protein expression. *P < 0.05. **G**, **H** Kaplan–Meier survival curve based on different expression levels of MLXIPL. **J** MLXIPL expression was significantly increased in HCC cells compared to L02 cells in mRNA level. ***P < 0.001. **I** MLXIPL protein was relatively overexpressed in HCC cells compared to L02 cells in protein level.

A violin plot revealed an elevated expression level of MLXIPL in the HCC cluster compared to the hepatocyte cluster (Fig. 5A). Based on the mRNA and protein level in the six patients used in this study, the expression level of MLXIPL in cancer tissues was generally higher than in normal liver tissues (Fig. 5B, C). Then, we performed qPCR and immunohistochemical (IHC) analyses of 68 primary HCC tumor specimens and their adjacent normal tissues. We observed that the levels of MLXIPL expression were substantially higher in the HCC samples than those in their adjacent normal tissues (Fig. 5D–F). The disease free and OS curve demonstrated that MLXIPL low-expressing group had better survival rate than that of the high-expressing group (Fig. 5G–H, *P* < 0.001 for both). The association between MLXIPL expression and clinical pathological characteristics was shown in Supplementary Table 4. The Cox proportional hazards model was used for univariate and multivariate analysis of OS and DFS (Supplementary Tables 5 and 6). In univariate analysis of OS and DFS, MLXIPL expression, tumor size, and TNM stage emerged as significant independent prognostic factors (Supplementary Table 5, *P* < 0.05 for all). Then, multivariate analysis revealed that MLXIPL expression, tumor size, and TNM stage (Supplementary Table 6, *P* < 0.05 for all) were independent prognostic factors for DFS. Vessel invasion and MLXIPL expression (Supplementary Table 6, *P* < 0.05 for all) were independent prognostic factors for OS. Thus, these results suggest that MLXIPL is likely involved in HCC progression and correlated with a poor prognosis in HCC.

Similar results appeared in immortalized liver cells and liver cancer cells. The expression of MLXIPL in HCC cell lines (SMMC-7721 and HepG2) was generally higher than that in immortalized liver cells L02 from mRNA and protein levels (Fig. 5I, J). These results indicate that high expression of MLXIPL is associated with poor prognosis of HCC.

### MLXIPL promotes HCC proliferation and inhibits its apoptosis in vitro

**Fig. 6:**
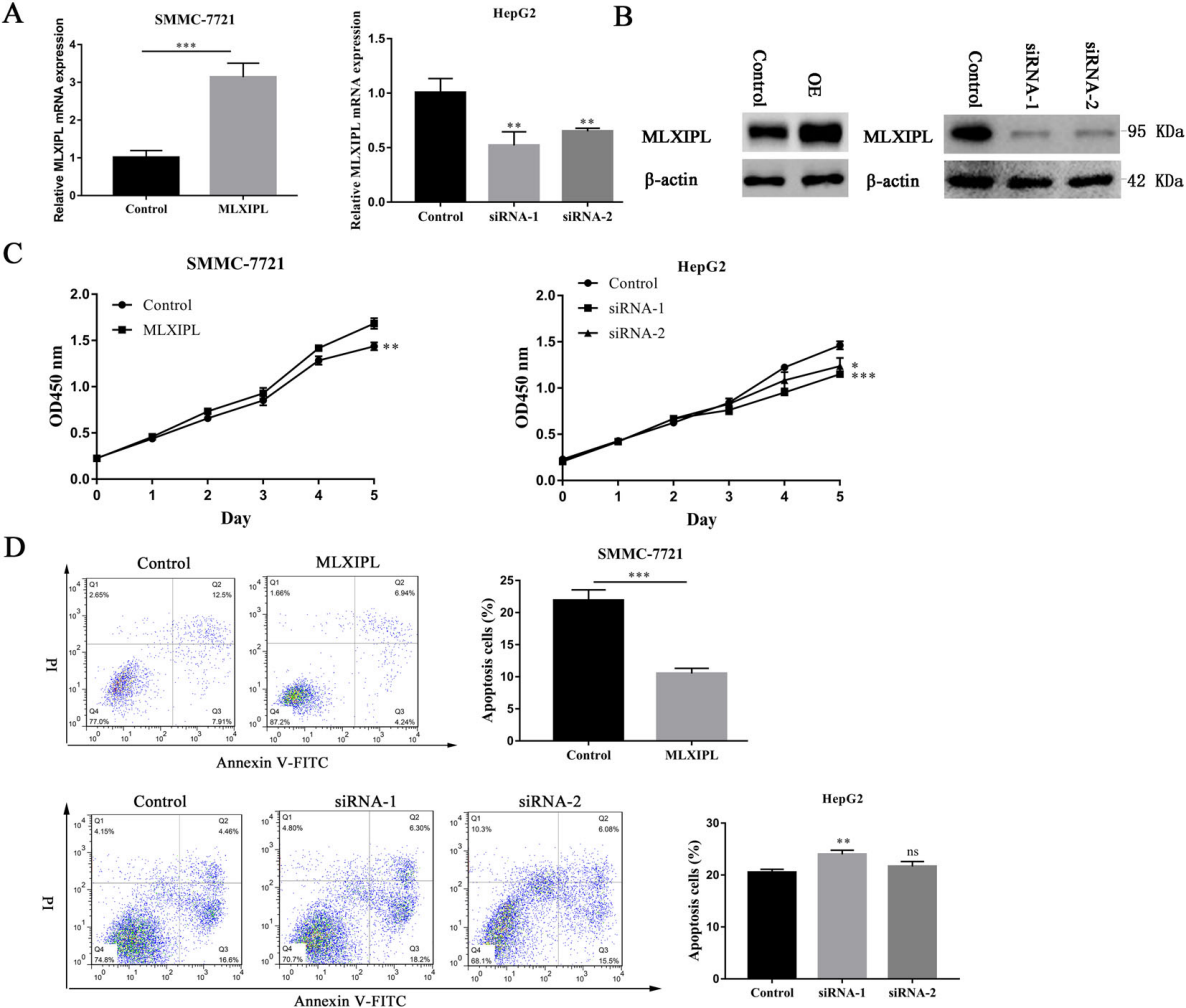
MLXIPL promotes HCC proliferation and inhibits apoptosis in vitro. **A**, **B** The transfection effect of overexpressed MLXIPL plasmids or MLXIPL siRNAs was measured by quantitative real-time PCR and western blotting. **P < 0.01; ***P < 0.001. **C** The proliferation ability in indicated cells was detected by the CCK8 assay after MLXIPL overexpression and knockdown separately in HCC cells. *P < 0.05; **P < 0.01; ***P < 0.001. **D** Apoptosis analysis in indicated cells was detected after MLXIPL overexpression and knockdown separately in HCC cells. Representative data are featured, presenting the population of living cells (Annexin V‑FITC−/PI−) in the left lower quadrant, early apoptotic cells (Annexin V‑FITC+/PI−) in right lower quadrant, late apoptotic cells (Annexin V‑FITC+/PI+) in the right upper quadrant and necrotic cells (Annexin V‑FITC−/PI+) in the left upper quadrant. ns *P* > 0.05; **P < 0.01; ***P < 0.001.

To explore the biological importance of the MLXIPL in HCC, MLXIPL siRNAs and overexpressed (OE) plasmids were transiently transfected into SMMC-7721 and HepG2 cells. The interference efficiency of the OE MLXIPL plasmids and MLXIPL siRNAs was verified by quantitative reverse transcription-quantitative polymerase chain reaction (PCR) and western blot analysis (Fig. 6A, B). To explore the effect on HCC proliferation and apoptosis, we performed the CCK8 and flow cytometry experiments. Transfected cells were used to examine their cell proliferative abilities, according to the CCK8 assay on days 1–5. The results showed that OE MLXIPL plasmids significantly promoted cell proliferation compared with the control, and MLXIPL siRNAs inhibited the proliferation of HCC cells (Fig. 6C). Quantitative apoptosis assay demonstrated that OE MLXIPL plasmids inhibited apoptosis compared with the control, and MLXIPL siRNAs promoted the apoptosis of HCC cells (Fig. 6D). These results indicate that MLXIPL promotes HCC proliferation and inhibits its apoptosis in vitro.

### MLXIPL elevates the activity of cell glycolysis in vitro

**Fig. 7:**
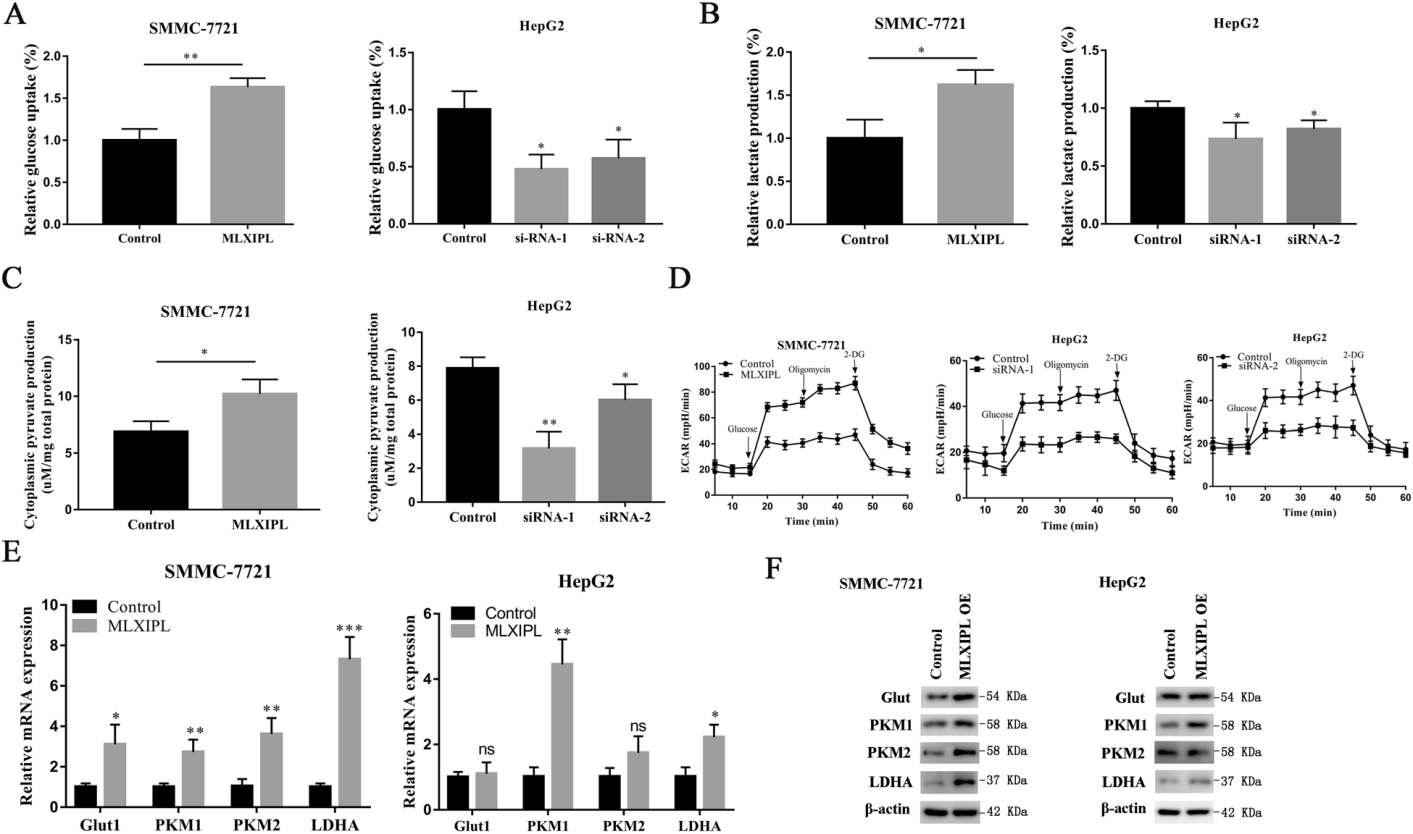
MLXIPL promotes HCC cell glycolysis in vitro. **A** SMMC-7721 and HepG2 cells transfected with overexpressed MLXIPL plasmids or MLXIPL siRNAs were cultured for 24 h for glucose uptake assays. *P < 0.05; **P < 0.01. **B** Analysis of the production of lactate in SMMC-7721 and HepG2 after transfected with overexpressed MLXIPL plasmids or MLXIPL siRNAs. *P < 0.05. **C** The transfected cells were lysed and the cytoplasmic levels of pyruvate were detected. *P < 0.05; **P < 0.01. **D** The overall glycolytic flux of transfected SMMC-7721 and HepG2 cells was analyzed by ECAR using seahorse instrument. **E**, **F** Quantitative real-time PCR and western blot were performed to analyze the levels changes of glycolytic key enzymes, when transfected with overexpressed MLXIPL plasmids in SMMC-7721 and HepG2 cells. ns *P* > 0.05; *P < 0.05; **P < 0.01; ***P < 0.001.

Studies have demonstrated that MLXIPL is responsible for increased levels of cell glycolysis^17,18^. The deletion of MLXIPL can decrease the gene expression of key glycolytic enzymes^19^. However, the glucose metabolism regulation of MLXIPL in HCC is not completely understood. We overexpressed or knocked down MLXIPL in HCC cells using OE plasmids or siRNAs, respectively. The results showed that OE MLXIPL significantly increased the glucose uptake and lactate production rates of HCC cell compared with control cells, whereas inhibiting MLXIPL decreased the glucose uptake and lactate production rates of HCC cell (Fig. 7A, B). Similar results appeared in kinetics of cytoplasmic pyruvate production experiment (Fig. 7C). In addition, MLXIPL also increased extracellular acidification rate (ECAR) in HCC cells, which indirectly reflected overall glycolytic flux (Fig. 7D). Usually, genes promote cancer cell glycolysis by upregulating the expression of key glycolysis enzymes. The results consistently revealed that most of the key glycolytic enzymes, glucose transporter type 1 (Glut1) mRNA, Pyruvate kinase muscle isozyme M1 and M2 (PKM1 and PKM2) mRNA, and labeling recombinant lactate dehydrogenase A(LDHA) mRNA and protein levels were upregulated upon MLXIPL overexpression in both SMMC-7721 and HepG2 cells (Fig. 7E, F). These results indicate that MLXIPL can increase glycolysis.

## Discussion

Sequencing technologies are now high throughput, enabling simultaneous sequencing of thousands to millions of genetic molecules^20,21^. Traditional sequencing approaches tend to obscure the underlying heterogeneity within phenotypically defined cell subpopulation. The results from single-cell sequencing facilitate comparisons between cells, depicting specimen heterogeneity, and enabling the discovery of novel subpopulations, which has obvious advantages^22^.

Previous studies have showed the heterogeneity in normal liver. MacParland et al.^10^ identified six distinct hepatocyte populations and two intrahepatic macrophage populations by scRNA-seq approaches. Zheng et al.^23^ determined the transcriptional profiles of liver immunological T cells with assembled T-cell receptor sequences, to identify 11 T-cell subsets^23^. In this study, we defined two hepatocyte subpopulations in hepatic tissues. The evidence indicated that hepatocyte heterogeneity exists in human liver samples, consistent with the results of MacParland et al.^10^. Our findings raised the possibility of a less localized and a more heterogeneous model of hepatocytes in the normal liver.

Heterogeneity is also a typical feature of tumors, which means that tumor cells undergo multiple proliferation and differentiation events, resulting in different tumor characteristics such as growth rate, invasiveness, metastatic capacity, and immune resistance, ultimately leading to the ineffectiveness of therapies and causing great challenges for treatment^24,25^. The genomic instability of HCC cells is an important reason for their high heterogeneity. HCC has a wide range of gene abnormal profiles and lacks clear characteristics of genetic change, which is different from other malignant tumors^26^. Gao et al.^27^ found that ten HCC patients displayed intra-tumor mutational heterogeneity and the mean occurrence of heterogeneous mutations was 39.7%. Bidkhori et al.^28^ identified three primary HCC subtypes and characterized heterogeneity in HCC by TCGA transcriptomics data sets. These findings were similar to our observations, in which we identified five subpopulations of single HCC cells that were different in genes expression levels, enriched pathways, and co-networks. We depicted t-SNE plots based on patient of origins, and the results showed a donor effect, which suggests an inter-tumor heterogeneity. In addition, each patient also contains a small amount of other cell subpopulations, suggesting an intra-tumor heterogeneity.

Cells can exhibit a series of different states (such as dynamic changes in gene expression, etc.) in various biological systems, and these states are transformed in a certain time sequence. When different cell subtypes are stimulated or disturbed externally or internally, a series of changes may occur in the expression of genes, presenting a series of state transitions. It may help to explain why one subtype can have different states. In this study, we noted that HCC0 has similar cellular trajectory to hepatocytes, which means they are in similar differentiation states. Studying the difference between the two clusters will provide new insights into liver carcinogenesis.

The liver is the foremost factory for the metabolism of nutrients, and various metabolic disorders occur in HCC patients^29,30^. In this study, we found that HCC3 subclone is enriched in metabolic disorder-related pathways. Pathway enrichment analyses revealed that metabolic disorder is responsible for HCC3 cell fate. Three major nutrient metabolism disorders, particularly lipids and lipoproteins disorders, were associated with the tumor development in HCC. Results of some biological studies can explain this phenomenon. For example, Lai et al.^31^ demonstrated that stearoyl-CoA desaturase synthesizes inhibited Wnt signaling, in part by acting on the stability of low-density lipoprotein receptor-related proteins 5 and 6. Lipid metabolism, identified by DEG analysis, has also been related to the inflection point in tumor evolution. UGT1A1, the most abundant UGT1A isoform, is a bilirubin-glucuronidating enzyme associated with the occurrence of HCC^32^. Hanczko et al.^33^ demonstrated that Taldo1-deficient mice spontaneously developed HCC preceded by the occurrence of steatosis, steatohepatitis, and cirrhosis, suggesting the pentose phosphate pathway is crucial for liver cancer.

Importantly, we noticed that several TF appeared to be particularly important in single cellular trajectory, including ONECUT1, DMBX1, RBPJL, HINFP, MLXIPL, ALX4, ONECUT1, and CEBPA/B. Researchers have verified that the expression of ONECUT1 was a suppressor gene in HCC^34,35^. Reebye et al.^36^ demonstrated that the upregulation of CEBPA can inhibit cell growth in HCC. In this study, we explored MLXIPL, a potential biomarker of HCC to better understand the molecular mechanisms of the differentiation to HCC. The results showed that MLXIPL was highly expressed in HCC tissues and cells, where it promoted the proliferation of HCC cells and inhibited its apoptosis, demonstrating that MLXIPL is an oncogene for prognosis in HCC.

The Warburg effect has been widely recognized as a hallmark of cancer^37^. Tumors are usually characterized by altered glucose flux from the tricarboxylic acid cycle to glycolysis. Increased glycolysis in HCC is often correlated with malignant biological behaviors^38^. It is acknowledged that glycolysis occurs in HCC. However, the mechanism driving glycolysis remains unknown. A study has demonstrated that MLXIPL is responsible for the increase of glycolytic mRNAs in response to excess carbohydrates. Elevated MLXIPL level is associated with the increased insulin sensitivity^39^. Hence, we focused on the effect of MLXIPL on aerobic glycolysis of HCC in subsequent studies. As expected, the results showed that MLXIPL is a positive regulator of glycolysis in HCC cells.

In summary, our scRNA sequencing workflow depicts a valuable framework for studying HCC. MLXIPL exhibits malignant biological behavior by activating HCC cell glycolysis. This study provides a better understanding toward the molecular mechanism of glycolysis in HCC, and highlights MLXIPL as a potential therapeutic target in HCC.

## Materials and methods

### Single-cell collection

Tissues used in this research were obtained from six patients pathologically diagnosed with HCC, at the Comprehensive Cancer Center of Shanghai General Hospital of Shanghai Jiao Tong University School of Medicine. The study was approved by the research institutional review board of our hospital, and all participants signed the informed consent. Tumor and para-tumor hepatic tissues were collected and immediately stored in sterile Dulbecco’s modified Eagle medium (DMEM) (Thermo Fisher Scientific), following the process flow shown in Supplementary Fig. 2D. Then, the tissues were transferred into pre-warmed DMEM medium containing 2 mg/ml collagenase P (Roche) and 10 U/µl DNase I (Roche). We gently pipetted the mixture and then digested for 60 min at 37 °C to fully release single cells. The cell suspension solution was filtered and centrifuged. The pellet was resuspended and 2 mM ethylene diamine tetraacetic acid in phosphate-buffered saline. We employed FACS to ensure the living cell selections. The majority of CD45+ leukocytes were removed using Dynabeads (Thermo Fisher Scientific, USA) from the cell suspension.

### ScRNA-seq library preparation and sequencing

ScRNA-seq was performed according to the manufacturer’s instructions of Smart-seq 2^40^ (Supplementary Fig. 2E). Reverse transcription was performed using Superscript reverse transcriptase (Takara) and locked TSO oligonucleotides (Exiqon). Full-length cDNA preamplification was conducted with 22 cycles of quantitative PCR amplification and HiFi-HotStart ReadyMix (KAPA Biosystems). Subsequently, Ampure XP beads (Beckman) were used for the purification. An Agilent high-sensitivity DNA chip was used to ensure the size and distribution of the cDNA library. Barcoded libraries were fragmented and tagged using a Nextera XT DNA preparation kit (Illumina). Then, we used reagents from the Nextera XT kit to amplify adapter-ligated fragments. Pooled libraries with unique N5-N7 barcodes were sequenced using a HiSeq 2500 instrument (Illumina) and single-end 50-bp read flow cells.

### ScRNA-seq data preprocessing and quality control

Fastq reads were initially filtered using Trimmomatic. The remaining clean reads were aligned to UCSC human genome 19 using Hisat 2. Next, we used Feature Counts software to quantify the expression of each gene, and counts were obtained for each sample. The expression level of each gene was converted to a transcript per million value. Then, the expression values were log-normalized. The strict filtration was then applied (Supplementary Table 2).

### Cell clustering and DEG analyses

We carried out robust clustering of unselected densities and determined that the cells in the same cluster acted as the same subtype, based on key gene mapping of different cell types using “Seurat” package (V3.1.2). To assign gene markers for single-cell clusters, DEGs were identified by calculating fold-change and *P* values between different groups using *t*-test method. We set a 1.5-fold cut-off of fold change and a false positive rate to *P* < 0.05, as the selection criteria. This was determined using the “stats” function in R. DEGs heatmaps were generated with heatmap R package (V1.0.12).

### Gene co-expression network (co-network) and pathway enrichment analyses

We constructed the network adjacency between genes, i and j, according to Pearson’s correlation between their expression profiles. Then, we obtained the co-network adjacency matrix by computing the correlation co-efficient. Next, we selected the genes with high correlations (0.8 or greater) to draw a hub-gene co-expression network graph using Cytoscape version 3.6.1.

The pathway enrichment analysis was based on GOBP and KEGG profiling by Metascape (http://metascape.org/) using *P* value cut-off 0.01.

### Single-cell trajectory analysis

We used diffusion mapping and Monocle to perform a pseudo-time analysis. Cells were chosen based on Seurat cluster identification results. Then, the key genes were obtained through *differentialGeneTest* Function in Monocle R package and filtered by the significance of *q* < 0.01 as cut-off. The TF from key genes were further selected by *dplyr* R package and ranked by *q* value to build a co-expression regulatory network as above described.

### Cell culture and transfection

L02, SMMC-7721, HepG2 cells were maintained in DMEM supplemented with 10% fetal bovine serum (Hyclone). All of the cell lines were from ATCC. Cells used in the experiments were authenticated by using short tandem repeat profiling.

HCC cells were plated at a density of 2 × 10^5^/well in a six-well plate 24 h before transfection. Transfection was performed using Lipofectamine 2000 transfection reagent (Thermo Fisher), according to the manufacturer’s protocol. Transfection efficiency was verified using quantitative reverse transcription-quantitative PCR (qRT-PCR) and western blotting.

### Quantitative reverse transcription-quantitative PCR (qRT-PCR)

Total RNA was extracted from transfected cells using the TRIzol reagent (Invitrogen), and the concentration was measured by NanoDrop1000 Spectrophotometer (Agilent). cDNA was reversed transcribed by the Superscript RT kit (TOYOBO) according to the manufacturer’s instructions. qRT-PCR amplification was performed using the SYBR Prime Script qRT-PCR kit (Takara). All quantization was normalized to the level of internal control GAPDH. Primer sequences are shown in Supplementary Table 7.

### Western blot analysis

Tissues and cells were lysed with a modified buffer, and western blotting was performed as described previously^41^. The primary antibodies were as follows: MLXIPL (Abcam, ab92809), GLUT1 (Abcam, ab115730), PKM1 (Abcam, ab116271), PKM2 (Abcam, ab137852), LDHA (Abcam, ab84716), and β-actin (Abbkine, A01011). And images were captured using an Amersham Imager 600 System (GE Healthcare).

### Immunohistochemistry

All the specimens embedded in paraffin blocks were cut at 3–4 μm and air-dried overnight. The tissue sections were deparaffinized, rehydrated, and subjected to heat-induced antigen retrieval with sodium citrate buffer (10 mM sodium citrate, 0.05% Tween-20 (pH 6.0)), which was followed by incubation with 3% hydrogen peroxide for 5 min to block endogenous peroxidase activity. Sections were then incubated with the appropriate primary antibody and were sequentially incubated with biotinylated goat anti-mouse IgG. For signal detection, the VECTASTAIN ABC kit (Vector Laboratories) was used according to the manufacturer’s instructions.

### CCK8 assay

In all, 1000 cells were plated in 96-well plates in 100 μl media. 10 μl Cell Counting Kit (CCK8) (Yeasen) solution was added into medium for 30 min before measuring absorbance at a wavelength of 450 nm by a microplate reader (Thermo Scientific) daily for continuous 5 days.

### Apoptosis analysis

Transfected cells were washed twice with ice-cold water, and stained with 5 μl of annexin V-FITC and 1 μl propidium iodide (PI, 1 mg/ml) for 15 min, and subjected to analysis on a flow cytometer (BD Biosciences).

### Glucose uptake and lactate production

SMMC-7721 and HepG2 cells transfected with MLXIPL OE plasmid or siRNAs were seeded in 12-well plates and incubated for 24 h in 37 °C incubator. For glucose uptake and lactate production assays, the culture medium was replaced with 500 μl DMEM. Glucose assay kit (Sigma) and lactate assay kit (Sigma) were applied according to the manufacturer’s instructions to detect cell lactate and glucose levels, respectively. All data were normalized by cell numbers.

### Cytoplasmic pyruvate assay

SMMC-7721 and HepG2 cells transfected with MLXIPL OE plasmid or siRNAs were seeded in 12-well plates and incubated for 24 h. After transfected cells lysed, the pyruvate levels in the cell lysates were measured by the pyruvate assay kit (Sigma, MAK071) according to the manufacturer’s instructions.

### Extracellular acidification rate (ECAR)

The Seahorse XF-96 Extracellular Flux Analyzer (Seahorse Bioscience) was used to measure the ECAR. ECAR was examined with a Seahorse XF glycolysis stress test kit according to the manufacturer’s protocols. In brief, cells (1 × 10^4^ cells/well) were seeded into a Seahorse XF-96 cell culture plate. After baseline measurements, glucose, oligomycin, and 2-DG were sequentially added into each well at the time points. ECAR data were assessed by Seahorse XF-96 Wave software and shown in mpH/min.

### Statistical analysis

All data were expressed as means ± standard error of the mean, and the statistical analysis was performed using GraphPad Prism v8.0. Comparisons between groups were performed using a one-way ANOVA or two-tailed Student’s *t* test. The Kaplan–Meier method was used to test the OS difference between two groups. A *P* value < 0.05 was considered statistically significant.

## Supplementary information

Supplementary Table. 1

Supplementary Table. 2

Supplementary Table. 3

Supplementary Table. 4

Supplementary Table. 5

Supplementary Table. 6

Supplementary Table. 7

Supplementary fugure 1

Supplementary figure 2

Supplementary figure 3

supplementary figure legend

## Data Availability

The data sets generated and/or analyzed during the current study are available in the GEO (GSE154906).
