# Supplementary material for: Single-cell analysis reveals the intra-tumor heterogeneity and identifies MLXIPL as a biomarker in the cellular trajectory of hepatocellular carcinoma
Source: Cell Death Discov. 2021 Jan 18;7:14. doi: 10.1038/s41420-021-00403-5 (PMC7814056; doi:10.1038/s41420-021-00403-5)
Supplement: Supplementary file 1 — Supplementary Table. 1 [file 41420_2021_403_MOESM1_ESM.docx]

**Supplementary Table 1. The Clinical Characteristics of the participating patients.**

|  | Patients ID | | | | | |
| --- | --- | --- | --- | --- | --- | --- |
| Clinical characteristics | PT1 | PT2 | PT3 | PT4 | PT5 | PT6 |
| Numbers of collected cells | 96 | 96 | 60 | 87 | 86 | 96 |
| Numbers of analysis cells | 88 | 88 | 50 | 61 | 50 | 68 |
| Age | 41 | 38 | 73 | 72 | 77 | 78 |
| Sex | Male | Male | Male | Male | Female | Male |
| Etiology |  |  |  |  |  |  |
| HBV | Yes | Yes | No | Yes | Yes | No |
| HCV | No | No | No | No | No | No |
| Alcohol | No | No | No | No | No | No |
| Aflatoxin B1 | No | No | No | No | No | No |
| Child-Pugh score | A | A | A | A | A | A |
| Stage (NCCN) | III | III | III | III | III | II |
| Tumor size (cm) | 3.5×3×3 | 7×7×5 | 11.5×11×7 | 10×5×4 | 7×3×4.8 | 4×3×4 |
| Tumor differentiation | Moderate | Moderate | Moderate | Moderate | Moderate | Moderate |
| Liver cirrhosis | Yes | Yes | No | Yes | Yes | No |
| MVI | MVI1 | MVI1 | MVI0 | MVI2 | MVI0 | MVI0 |
| AFP (ng/ml) | 4.52 | >1210 | 207 | 6.3 | >1210 | 4.64 |
| CEA (ng/ml) | 6.94 | 1.44 | 5.4 | 4.3 | 2.5 | 2.86 |
