# Supplementary material for: Single-cell analysis reveals the intra-tumor heterogeneity and identifies MLXIPL as a biomarker in the cellular trajectory of hepatocellular carcinoma
Source: Cell Death Discov. 2021 Jan 18;7:14. doi: 10.1038/s41420-021-00403-5 (PMC7814056; doi:10.1038/s41420-021-00403-5)
Supplement: Supplementary file 2 — Supplementary Table. 2 [file 41420_2021_403_MOESM2_ESM.docx]

**Supplementary Table 2. The basic filtering parameter of the HCC single cells of detected.**

|  | Patients ID | | | | | |
| --- | --- | --- | --- | --- | --- | --- |
| Parameter | PT01 | PT02 | PT03 | PT04 | PT05 | PT06 |
| Number of single cells collected | 96 | 96 | 60 | 87 | 86 | 96 |
| Read Counts more than 10000 | 90 | 92 | 58 | 85 | 83 | 76 |
| Number of genes detected more than 2000 | 88 | 92 | 58 | 85 | 76 | 68 |
| Number of mitochondrial genes proportion less than 20% | 88 | 88 | 50 | 61 | 50 | 68 |
| Total qualified cell count | 88 | 88 | 50 | 61 | 50 | 68 |
| Percent of cell after filtration | 92% | 92% | 93% | 70% | 58% | 71% |
