# Supplementary material for: Single-cell analysis reveals the intra-tumor heterogeneity and identifies MLXIPL as a biomarker in the cellular trajectory of hepatocellular carcinoma
Source: Cell Death Discov. 2021 Jan 18;7:14. doi: 10.1038/s41420-021-00403-5 (PMC7814056; doi:10.1038/s41420-021-00403-5)
Supplement: Supplementary file 3 — Supplementary Table. 3 [file 41420_2021_403_MOESM3_ESM.docx]

**Supplementary Table. 3 The regulatory relationship between evolutionary related transcription factors and their regulated genes**

| Transcription factor | Gene | Correlation | P value |
| --- | --- | --- | --- |
| MLXIPL | RBP4 | 0.630100179 | 6.47E-38 |
| CEBPA | EFNA1 | 0.628944133 | 9.63E-38 |
| ONECUT1 | GJB1 | 0.618687439 | 3.04E-36 |
| CEBPB | RBP4 | 0.618223561 | 3.54E-36 |
| CEBPA | RBP4 | 0.616180533 | 6.93E-36 |
| CEBPB | GPX2 | 0.611847175 | 2.83E-35 |
| MLXIPL | A1BG | 0.607259214 | 1.23E-34 |
| CEBPB | IGFBP1 | 0.606620009 | 1.50E-34 |
| CEBPB | DCXR | 0.604679758 | 2.77E-34 |
| CEBPB | APCS | 0.604656185 | 2.80E-34 |
| CEBPA | C1orf85 | 0.603629896 | 3.86E-34 |
| CEBPB | TMEM176A | 0.601918723 | 6.58E-34 |
| CEBPB | POLD4 | 0.600273871 | 1.10E-33 |
| CEBPA | GJB1 | 0.597704603 | 2.42E-33 |
| CEBPA | APOC3 | 0.593664751 | 8.28E-33 |
| CEBPA | FABP1 | 0.593359184 | 9.08E-33 |
| ONECUT1 | FABP1 | 0.593034645 | 1.00E-32 |
| ONECUT1 | SNORD81 | 0.590034292 | 2.46E-32 |
| CEBPA | AMBP | 0.58912933 | 3.23E-32 |
| ONECUT1 | TMEM176A | 0.588639573 | 3.73E-32 |
| ONECUT1 | ITM2B | 0.587826579 | 4.75E-32 |
| CEBPB | GJB1 | 0.58739122 | 5.40E-32 |
| MLXIPL | SERPINF2 | 0.58699504 | 6.08E-32 |
| MLXIPL | APOC3 | 0.586653788 | 6.72E-32 |
| CEBPB | SERPINF2 | 0.585319152 | 9.95E-32 |
| CEBPA | SDF2L1 | 0.584891732 | 1.13E-31 |
| ONECUT1 | MRPL24 | 0.583915657 | 1.50E-31 |
| CEBPA | A1BG | 0.582251111 | 2.44E-31 |
| ONECUT1 | RBP4 | 0.581456346 | 3.07E-31 |
| CEBPB | HIST2H2AC | 0.580955526 | 3.55E-31 |
| CEBPA | APOA1 | 0.57939818 | 5.57E-31 |
| MLXIPL | MST1 | 0.5779705 | 8.39E-31 |
| CEBPB | NDUFB7 | 0.576835552 | 1.16E-30 |
| CEBPB | SERPINA3 | 0.575278572 | 1.81E-30 |
| JUND | RBP4 | 0.57509925 | 1.90E-30 |
| MLXIPL | CFB | 0.5749785 | 1.97E-30 |
| CEBPB | APOC3 | 0.574647927 | 2.16E-30 |
| ONECUT1 | COX8A | 0.573803213 | 2.74E-30 |
| CEBPB | RARRES2 | 0.572395568 | 4.08E-30 |
| CEBPA | PGRMC1 | 0.572353603 | 4.13E-30 |
| ONECUT1 | SERPINA6 | 0.572131887 | 4.39E-30 |
| CEBPB | CST3 | 0.571992604 | 4.57E-30 |
| CEBPA | ASGR1 | 0.570995618 | 6.04E-30 |
| MLXIPL | AGXT | 0.570306707 | 7.32E-30 |
| CEBPB | EID1 | 0.570060596 | 7.84E-30 |
| ONECUT1 | PCK1 | 0.569407408 | 9.40E-30 |
| ONECUT1 | EFNA1 | 0.568598146 | 1.18E-29 |
| CEBPB | AGXT | 0.568320367 | 1.27E-29 |
| CEBPB | FABP1 | 0.567649274 | 1.53E-29 |
| CEBPB | DUSP23 | 0.567084467 | 1.79E-29 |
| DMBX1 | RPS13 | -0.13778606 | 0.012228823 |
| DMBX1 | PGRMC1 | -0.13783793 | 0.012195934 |
| HINFP | RPS9 | -0.13827517 | 0.011921765 |
| ZNF35 | TXNIP | -0.13836949 | 0.011863339 |
| RBPJL | GPX2 | -0.13842446 | 0.011829406 |
| DMBX1 | AKR1C3 | -0.13919774 | 0.011361057 |
| RBPJL | NEAT1 | -0.13920122 | 0.011358982 |
| RBPJL | RPS5 | -0.14172096 | 0.009944777 |
| HINFP | TMSB10 | -0.14179861 | 0.009903796 |
| DMBX1 | MST1P9 | -0.14220232 | 0.009693119 |
| DMBX1 | POLD4 | -0.14238092 | 0.009601192 |
| HINFP | SAT2 | -0.14274562 | 0.009415877 |
| DMBX1 | NDUFA2 | -0.14291697 | 0.009329911 |
| DMBX1 | DCPS | -0.14462284 | 0.008511359 |
| RBPJL | SOD1 | -0.14471065 | 0.008471011 |
| DMBX1 | FABP1 | -0.14506234 | 0.008311107 |
| HINFP | VTN | -0.14541742 | 0.008152393 |
| HINFP | NDUFA13 | -0.14548054 | 0.008124462 |
| DMBX1 | RPS9 | -0.14550211 | 0.008114941 |
| DMBX1 | S100A10 | -0.14557077 | 0.008084689 |
| ALX4 | SAT1 | -0.14581938 | 0.007975989 |
| HINFP | EID1 | -0.14700057 | 0.007477138 |
| DMBX1 | HNRNPA2B1 | -0.14753446 | 0.007260942 |
| DMBX1 | RBP4 | -0.14904233 | 0.006680191 |
| DMBX1 | ID2B | -0.15065541 | 0.006105254 |
| RBPJL | RPL11 | -0.15094944 | 0.006005371 |
| DMBX1 | CFB | -0.15153607 | 0.005810459 |
| HINFP | GPX2 | -0.15243514 | 0.005522743 |
| HINFP | GNB2L1 | -0.15375005 | 0.005124971 |
| ZNF132 | NCL | -0.15383765 | 0.005099411 |
| ALX4 | AKR1C2 | -0.15472526 | 0.004846817 |
| DMBX1 | NEAT1 | -0.15567492 | 0.004589092 |
| DMBX1 | RPL37 | -0.15643563 | 0.004391628 |
| DMBX1 | RPL13A | -0.15813745 | 0.003977362 |
| DMBX1 | AKR1C2 | -0.15887048 | 0.003810068 |
| HINFP | AKR1C1 | -0.15957299 | 0.00365574 |
| DMBX1 | ETFB | -0.16009529 | 0.003544693 |
| DMBX1 | APOA2 | -0.16257116 | 0.00305873 |
| HINFP | PAH | -0.16284501 | 0.003008873 |
| HINFP | COX8A | -0.1629507 | 0.00298983 |
| DMBX1 | TPT1 | -0.16342874 | 0.002905056 |
| DMBX1 | AGXT | -0.16522872 | 0.002605086 |
| ALX4 | APOB | -0.16901483 | 0.002064104 |
| RBPJL | HSPA5 | -0.16987118 | 0.001956942 |
| DMBX1 | RPL36 | -0.1699633 | 0.001945722 |
| DMBX1 | CFHR1 | -0.17036689 | 0.001897258 |
| HINFP | AGT | -0.17112028 | 0.001809729 |
| DMBX1 | HPN | -0.17468656 | 0.001443403 |
| DMBX1 | HSPB1 | -0.18701627 | 0.000638751 |
| RBPJL | FTL | -0.19571693 | 0.000348181 |
