# Supplementary material for: Single-cell analysis reveals the intra-tumor heterogeneity and identifies MLXIPL as a biomarker in the cellular trajectory of hepatocellular carcinoma
Source: Cell Death Discov. 2021 Jan 18;7:14. doi: 10.1038/s41420-021-00403-5 (PMC7814056; doi:10.1038/s41420-021-00403-5)
Supplement: Supplementary file 4 — Supplementary Table. 4 [file 41420_2021_403_MOESM4_ESM.docx]

**Supplementary Table 4. Correlation between MLXIPL expression and clinicopathological features**

| Parameters | Total  N=68 | MLXIPL protein expression | | p value |
| --- | --- | --- | --- | --- |
|  |  | Negative 30(44.1%) | Positive 38(55.9%) |  |
| Age, years |  |  |  | **0.008** |
| <60 | 33 | 20 (66.7%) | 13 (34.2%) |  |
| ≥60 | 35 | 10 (33.3%) | 25 (65.8%) |  |
| Gender |  |  |  | 0.943 |
| Female | 7 | 3 (10.0%) | 4 (90.0%) |  |
| Male | 61 | 27 (10.3%) | 34 (89.7%) |  |
| Vessel invasion |  |  |  | **0.001** |
| No | 27 | 20 (66.7%) | 7 (21.1%) |  |
| Yes | 41 | 10 (39.7%) | 31 (81.6%) |  |
| Tumor number |  |  |  | **0.010** |
| single | 29 | 18 (60.0%) | 11 (28.9%) |  |
| multiple | 39 | 12 (40.0%) | 27 (57.4%) |  |
| Tumor differentiation |  |  |  | 0.543 |
| I-II | 30 | 12 (40.0%) | 18 (47.4%) |  |
| III-IV | 38 | 18 (60.0%) | 20 (52.8%) |  |
| Tumor size |  |  |  | **0.001** |
| <5cm | 33 | 22 (73.3%) | 11 (28.9%) |  |
| >5cm | 35 | 8 (26.7%) | 27 (71.1%) |  |
| HBsAg |  |  |  | **0.001** |
| - | 24 | 19 (63.3%) | 5 (13.2%) |  |
| + | 44 | 11 (36.7%) | 33 (86.8%) |  |
| Liver cirrhosis |  |  |  | **0.001** |
| No | 35 | 22 (73.3%) | 13 (34.2%) |  |
| Yes | 33 | 8 (26.7%) | 25 (65.8%) |  |
| Serum AFP ng/ml |  |  |  | 0.276 |
| ≤20 | 29 | 15 50.0%) | 14 (36.8%) |  |
| ＞20 | 39 | 15 (50.0%) | 24 (63.2%) |  |
| TNM |  |  |  | **0.001** |
| I-II | 34 | 25 (83.3%) | 9 (23.7%) |  |
| III-IV | 34 | 5 (16.7%) | 29 (76.3%) |  |
|  |  |  |  |  |
