# Supplementary material for: Single-cell analysis reveals the intra-tumor heterogeneity and identifies MLXIPL as a biomarker in the cellular trajectory of hepatocellular carcinoma
Source: Cell Death Discov. 2021 Jan 18;7:14. doi: 10.1038/s41420-021-00403-5 (PMC7814056; doi:10.1038/s41420-021-00403-5)
Supplement: Supplementary file 5 — Supplementary Table. 5 [file 41420_2021_403_MOESM5_ESM.docx]

| Factors | DFS | | |  |  | OS | |
| --- | --- | --- | --- | --- | --- | --- | --- |
|  | HR (95% CI) | P value | |  |  | HR (95% CI) | P value |
| Age, years |  | | **0.003** |  |  |  | 0.160 |
| <60 |  | |  |  |  |  |  |
| ≥60 | 2.642 (1.392-5.016) | |  |  |  | 1.611 (0.828 -3.136) |  |
| Gender |  | | 0.543 |  |  |  | 0.245 |
| Male | 1.331 (0.530-3.341) | |  |  |  | 2.340 (0.559-9.800) |  |
| Female |  | |  |  |  | - |  |
| Vessel invasion |  | | **0.002** |  |  |  | **0.000** |
| No |  | |  |  |  |  |  |
| Yes | 2.821 (1.442-5.519) | |  |  |  | 4.530 (2.058-9.841) |  |
| Tumor number |  | | 0.331 |  |  |  | 0.262 |
| single |  | |  |  |  |  |  |
| multiple | 1.361 (0.731-2.533) | |  |  |  | 1.469 (0.750-2.875) |  |
| Tumor differentiation |  | | 0.257 |  |  |  | 0.346 |
| I-II |  | |  |  |  |  |  |
| III-IV | 1.417 (0.775-2.589) | |  |  |  | 1.361 (0.717-2.583) |  |
| Tumor size |  | | **0.003** |  |  |  | **0.002** |
| <5cm |  | |  |  |  |  |  |
| >5cm | 2.702 (1.418-5.147) | |  |  |  | 2.933 (1.472-5.845) |  |
| HBsAg |  | | **0.005** |  |  |  | 0.063 |
| - |  | |  |  |  |  |  |
| + | 2.789 (1.365-5.737) | |  |  |  | 2.063 (0.962-4.306) |  |
| Liver cirrhosis |  | | 0.060 |  |  |  | 0.478 |
| No |  | |  |  |  |  |  |
| Yes | 1.786 (0.975-3.270) | |  |  |  | 1.264 (0.662-2.414) |  |
| Serum AFP ng/ml |  | | 0.484 |  |  |  | 0.438 |
| ≤20 |  | |  |  |  |  |  |
| ＞20 | 1.239 (0.680-2.258) | |  |  |  | 1.250 (0.711-2.197) |  |
| TNM |  | | **0.008** |  |  |  | **0.001** |
| I-II |  | |  |  |  |  |  |
| III-IV | 2.366 (1.253-4.467) | |  |  |  | 3.111 (1.575-6.145) |  |
| MLXIPL expression |  | | **0.000** |  |  |  | **0.000** |
| Negative |  | |  |  |  |  |  |
| Positive | 6.969 (3.196-15.191) | |  |  |  | 4.754 (2.089-10.819) |  |

**Supplementary Table. 5 Univariate analysis for disease-free survival (DFS) and overall survival (OS)**
