# Supplementary material for: Single-cell analysis reveals the intra-tumor heterogeneity and identifies MLXIPL as a biomarker in the cellular trajectory of hepatocellular carcinoma
Source: Cell Death Discov. 2021 Jan 18;7:14. doi: 10.1038/s41420-021-00403-5 (PMC7814056; doi:10.1038/s41420-021-00403-5)
Supplement: Supplementary file 6 — Supplementary Table. 6 [file 41420_2021_403_MOESM6_ESM.docx]

**Supplementary Table. 6 Multivariate analysis for DFS and OS**

| Factors | DFS | | |  |  | OS | |
| --- | --- | --- | --- | --- | --- | --- | --- |
|  | HR (95% CI) | P value | |  |  | HR (95% CI) | P value |
| Age, years |  | | 0.123 |  |  |  |  |
| <60 |  | |  |  |  |  |  |
| ≥60 | 1.788 (0.855-3.739) | |  |  |  |  |  |
| Vessel invasion |  | | 0.640 |  |  |  | **0.046** |
| No |  | |  |  |  |  |  |
| Yes | 1.207 (0.549-2.653) | |  |  |  | 2.461 (1.017-5.956) |  |
| Tumor size |  | | **0.018** |  |  |  | 0.488 |
| <5cm |  | |  |  |  |  |  |
| >5cm | 4.491 (1.287-15.668) | |  |  |  | 1.700 (0.380-7.604) |  |
| HBsAg |  | | 0.316 |  |  |  |  |
| - |  | |  |  |  |  |  |
| + | 1.675 (0.611-4.592) | |  |  |  |  |  |
| TNM |  | | **0.028** |  |  |  | 0.712 |
| I-II |  | |  |  |  |  |  |
| III-IV | 5.024 (1.186-21.288) | |  |  |  | 1.349 (0.276-6.591) |  |
| MLXIPL expression |  | | **<0.001** |  |  |  | **0.032** |
| Negative |  | |  |  |  | 3.125 (1.103-8.859) |  |
| Positive | 7.716 (2.747-21.668) | |  |  |  |  |  |
