# Supplementary material for: Single-cell analysis reveals the intra-tumor heterogeneity and identifies MLXIPL as a biomarker in the cellular trajectory of hepatocellular carcinoma
Source: Cell Death Discov. 2021 Jan 18;7:14. doi: 10.1038/s41420-021-00403-5 (PMC7814056; doi:10.1038/s41420-021-00403-5)
Supplement: Supplementary file 7 — Supplementary Table. 7 [file 41420_2021_403_MOESM7_ESM.docx]

**Supplementary Table 7. Sequence of PCR primers.**

| Name | Forward (5’-3’) | Reverse (5’-3’) |
| --- | --- | --- |
| Glut1 | ACCACCTCACTCCTGTTACTT ACCT | ATCCAAACCTCCTACCCTCAATCCA |
| PKM1 | CGAGCCTCAAGTCACTCCAC | GTGAGCAGACCTGCCAGACT |
| PKM2 | GTGCCGCCTGGACATTGATTCA | AGTTCAGACGAGCCACATTCATTCC |
| LDHA | TAGGCTACAACAGGATTCTAGGTGGAG | GTCAGAGGTGGCAGAACTATTTC |
| MLXIPL | GCAGTATCGACCCCACAC | TCCAGATGGCGTTGTTCA |
| GAPDH | AGCCTCAAGATCATCAGCAATGCC | TGTGGTCATGAGTCCTTCCACGAT |
