# Supplementary figures and images for: Single-cell analysis reveals the intra-tumor heterogeneity and identifies MLXIPL as a biomarker in the cellular trajectory of hepatocellular carcinoma
Source: Cell Death Discov. 2021 Jan 18;7:14. doi: 10.1038/s41420-021-00403-5 (PMC7814056; doi:10.1038/s41420-021-00403-5)

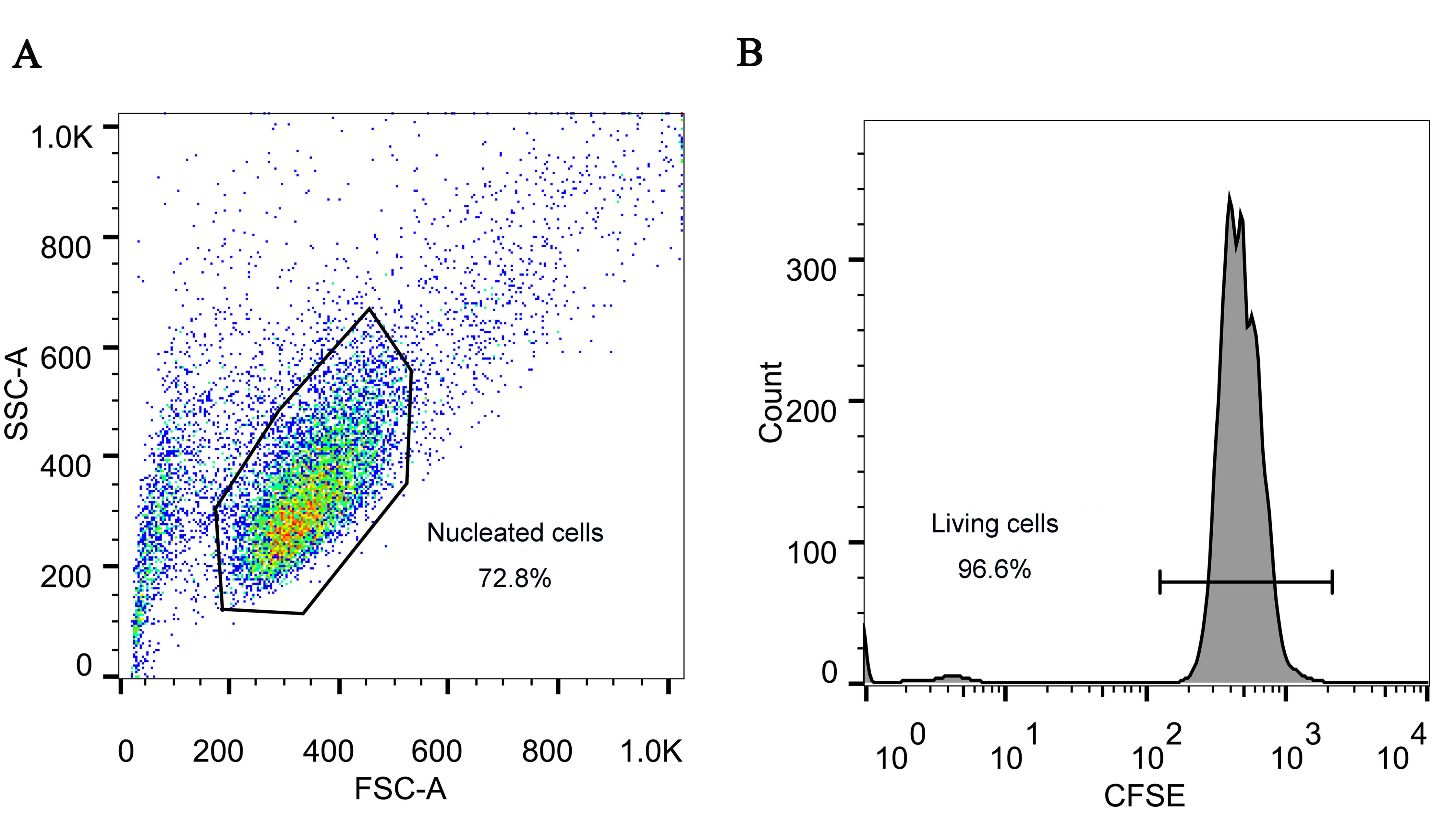

Supplement: Supplementary file 8 — Supplementary fugure 1 [file 41420_2021_403_MOESM8_ESM.tif]

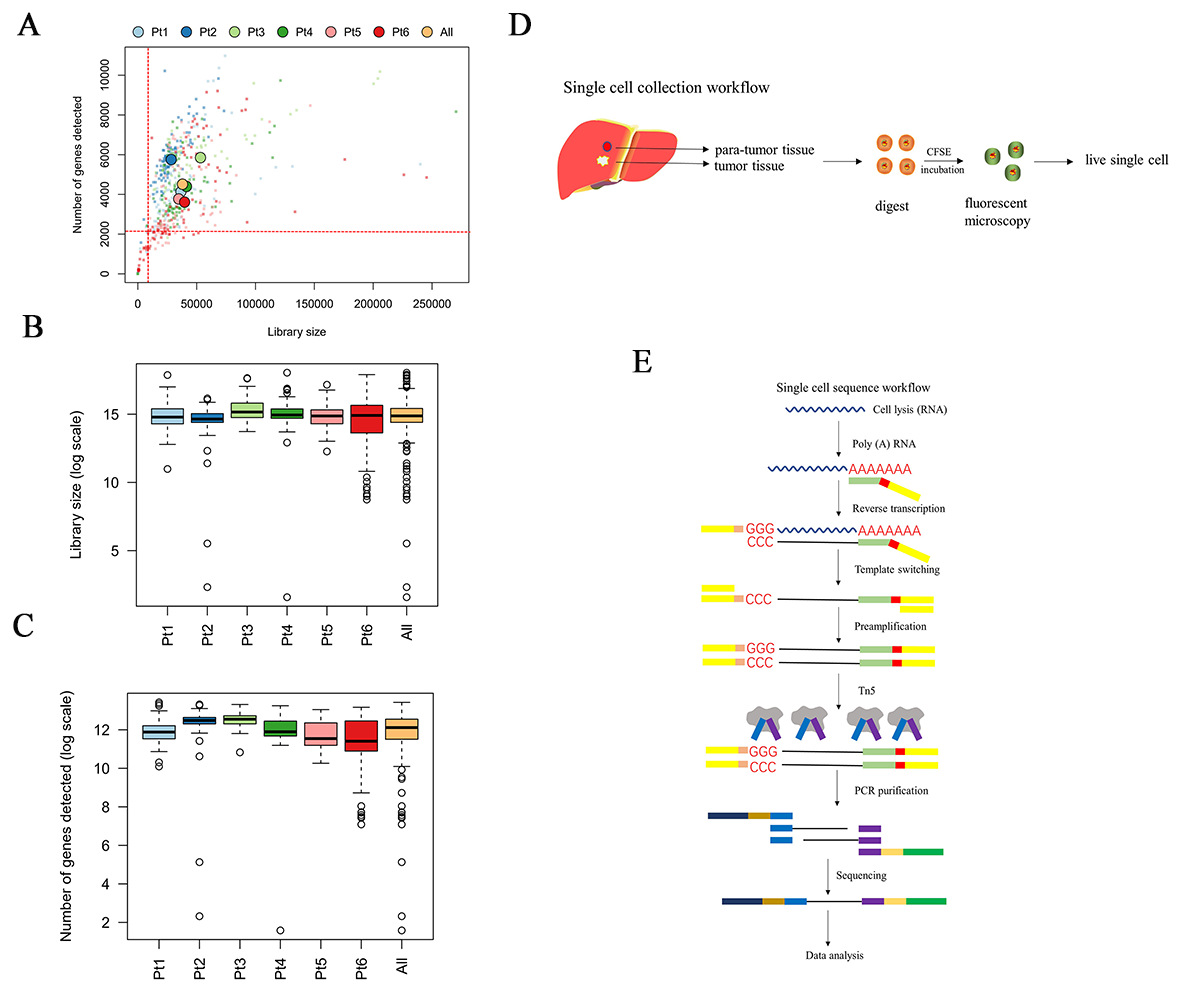

Supplement: Supplementary file 9 — Supplementary figure 2 [file 41420_2021_403_MOESM9_ESM.tif]

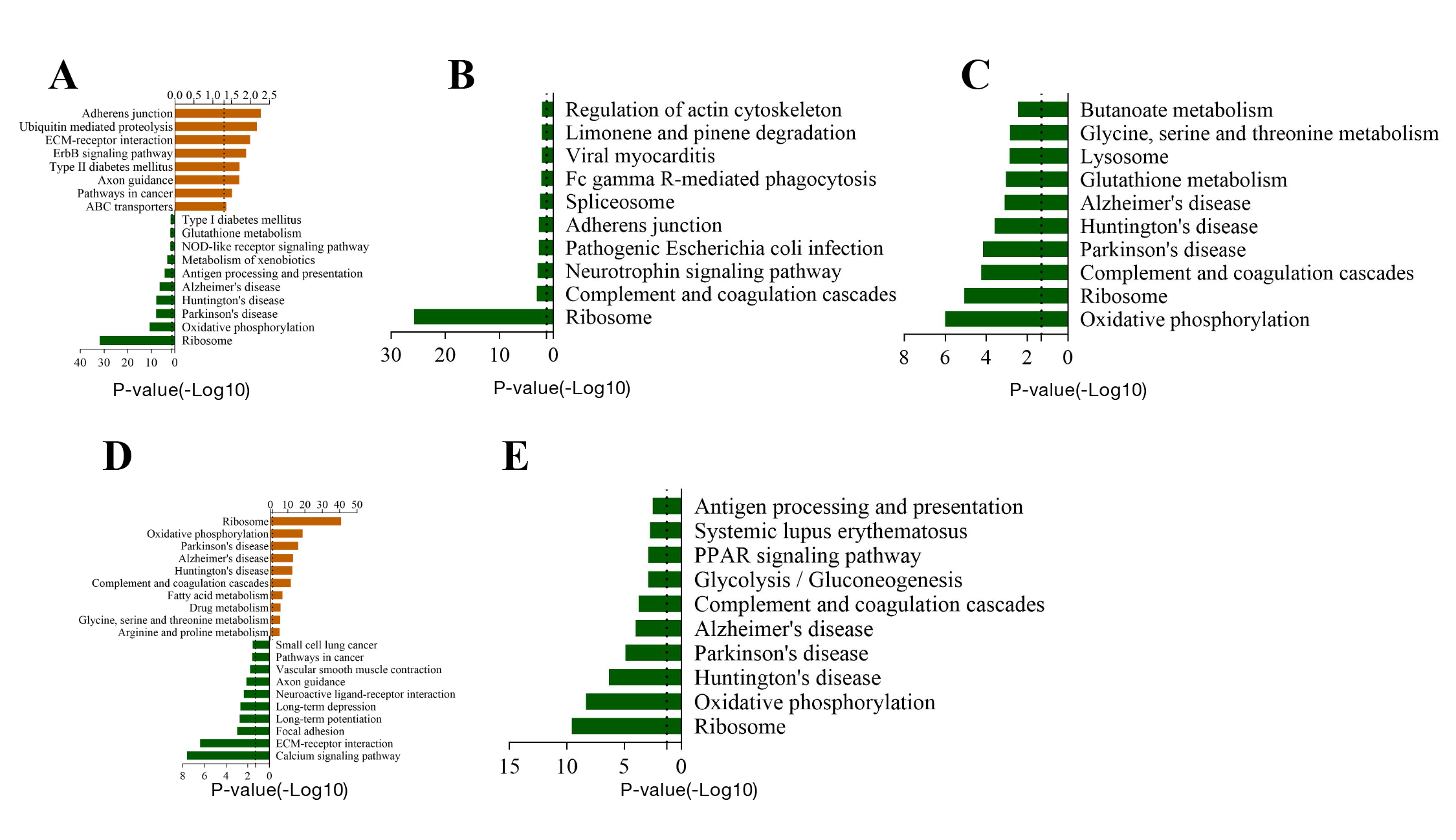

Supplement: Supplementary file 10 — Supplementary figure 3 [file 41420_2021_403_MOESM10_ESM.tif]
