# Supplementary material for: Single-cell analysis reveals the intra-tumor heterogeneity and identifies MLXIPL as a biomarker in the cellular trajectory of hepatocellular carcinoma
Source: Cell Death Discov. 2021 Jan 18;7:14. doi: 10.1038/s41420-021-00403-5 (PMC7814056; doi:10.1038/s41420-021-00403-5)
Supplement: Supplementary file 11 — supplementary figure legend [file 41420_2021_403_MOESM11_ESM.docx]

**Supplementary Fig. 1** Identification of living single cells by FACS. **a.** The example depicted 72.8% nucleated cells from one patient identified by FACS and **b** a 96.6% living cell rate determined after staining by CFSE.

**Supplementary Fig. 2** ScRNA-seq workflow of single cells from HCC tissues. **a.** Viable cells were filtered from single cell libraries having a minimum library size of 10000 and minimum number of 2000 genes detected. **b and c.** Box diagram showed the library size and number of genes detected in each patient. **d.** Workflow of dissociation for HCC tumor and para-tumor tissues to obtain single cells. **e.** ScRNA-seq workflow using the Smart-seq 2 method.

**Supplementary Fig. 3** KEGG functional analysis of HCC subclones. **a.** KEGG functional analysis of HCC 0 (cluster 0). **b.** KEGG functional analysis of HCC 1 (cluster 1). **c.** KEGG functional analysis of HCC 2 (cluster 2). **d.** KEGG functional analysis of HCC 3 (cluster 3). **e.** KEGG functional analysis of HCC 4 (cluster 4).

**Supplementary Table 1.** The Clinical Characteristics of the participating patients.

**Supplementary Table 2.** The basic filtering parameter of the HCC single cells of detected.

**Supplementary Table 3.** The regulatory relationship between evolutionary related transcription factors and their regulated genes.

**Supplementary** **Table 4.** Correlation between MLXIPL expression and clinicopathological features

**Supplementary** **Table 5.** Univariate analysis for disease-free survival (DFS) and overall survival (OS)

**Supplementary** **Table 6.** Multivariate analysis for DFS and OS

**Supplementary Table 7.** Sequence of PCR primers.
